# Parasitological, Molecular, and Epidemiological Investigation of *Cryptosporidium* Infection Among Cattle and Buffalo Calves From Assiut Governorate, Upper Egypt: Current Status and Zoonotic Implications

**DOI:** 10.3389/fvets.2022.899854

**Published:** 2022-06-17

**Authors:** Ehab Kotb Elmahallawy, Hesham A. Sadek, Dina Aboelsoued, Maha A. Aloraini, Abdulsalam A. M. Alkhaldi, Salma M. Abdel-Rahman, Hanna Y. Bakir, Mohsen I. Arafa, Ehssan Ahmed Hassan, Elzahara Elbaz, Eman A. A. Hassanen, Fatma A. El-Gohary, Ahmed Gareh

**Affiliations:** ^1^Department of Zoonoses, Faculty of Veterinary Medicine, Sohag University, Sohag, Egypt; ^2^Animal Health Research Institute, Giza, Egypt; ^3^Department of Parasitology and Animal Diseases, National Research Centre, Veterinary Research Institute, Giza, Egypt; ^4^Department of Biology, Faculty of Science and Humanities, Shaqra University, Shaqra, Saudi Arabia; ^5^Biology Department, College of Science, Jouf University, Sakakah, Saudi Arabia; ^6^Department of Medical Parasitology, Faculty of Medicine, Assiut University, Asyut, Egypt; ^7^Biology Department, College of Science and Humanities, Prince Sattam Bin Abdul Aziz University, Alkharj, Saudi Arabia; ^8^Department of Zoology, Faculty of Science, Suez Canal University, Ismailia, Egypt; ^9^Department of Internal Medicine and Infectious Diseases, Faculty of Veterinary Medicine, Mansoura University, Mansoura, Egypt; ^10^Department of Parasitology, Faculty of Veterinary Medicine, Zagazig University, Sharkia, Egypt; ^11^Department of Hygiene and Zoonoses, Faculty of Veterinary Medicine, Mansoura University, Mansoura, Egypt; ^12^Department of Parasitology, Faculty of Veterinary Medicine, Aswan University, Aswan, Egypt

**Keywords:** *Cryptosporidium* spp., parasitology, epidemiology, cattle, buffalo, calves, Egypt

## Abstract

Details about the epidemiological patterns and real contributions of different reservoir animals in maintaining the transmission cycle of *Cryptosporidium spp*. in Upper Egypt remain lacking. This study was designed to investigate the occurrence of *Cryptosporidium* spp. in cattle and buffalo (*n* = 608) from Upper Egypt. The parasite for the resulting positive samples by fecal examination was molecularly identified using nested PCR targeting the small subunit rRNA. Moreover, several explanatory variables, including animals' age, sex, condition, seasonal variations, were examined to describe the epidemiological pattern of the disease. Interestingly, the fecal examination revealed that 33.55% (204/608) of the animals under study were infected with *Cryptosporidium*, including 38.27% among cattle and 28.16% among buffalo. The parasite was molecularly identified using nested PCR, and their amplicons were identified in almost all fecal samples using microscopy (202/204). According to age as an individual variable factor, the infection rates of *Cryptosporidium* spp. in cattle calves with ages of <1, 1–3, and >3 months were 39.13, 34.04, and 54.54%, respectively. Meanwhile, in buffalo calves, the occurrence rates were 28.57, 27.27, and 29.41%, respectively. Regarding sex, female cattle calves were more susceptible to *Cryptosporidium* infection (51.28%) than males (26.19%) (*p* < 0.05), whereas male buffalo calves had a higher infection rate (32.25%) than females (25%). According to seasonal variations, the infection rates of *Cryptosporidium* spp. in cattle calves during spring, summer, autumn, and winter were 42.11, 30.43, 30, and 52.63%, respectively. In contrast, lower infection rates of 30, 21.42, 23.52, and 35% were reported in buffalo calves during spring, summer, autumn, and winter, respectively. The rate of infection was 45.16% in diarrheic cattle calves and 15.78% in non-diarrheic ones (*p* < 0.05). Meanwhile, the infection rate was 33.96% in diarrheic buffalo calves and 11.11% in non-diarrheic ones (*p* < 0.05). This study reported a higher occurrence of *Cryptosporidium* infection among the animals under study and revealed that buffalos and cattle can contribute to maintaining the transmission cycle of this zoonotic parasite in Upper Egypt.

## Introduction

Cryptosporidiosis is a major diarrheal disease, which is caused by the protozoan parasites of the genus *Cryptosporidium*. These organisms are one of the most prevalent parasites that infect domesticated cattle and buffalos (1). The genus *Cryptosporidium* consists of eukaryotic protozoal intracellular parasites and is classified as a member of the phylum Apicomplexa (1, 2). The four major *Cryptosporidium* species that infect cattle are *Cryptosporidium parvum, Cryptosporidium bovis, Cryptosporidium ryanae*, and *Cryptosporidium andersoni* (3). Considering its epidemiological profile, *Cryptosporidium* infects various livestock, resulting in significant economic losses (4).

Infection with *Cryptosporidium* is initiated when viable oocysts are ingested by a susceptible host through the fecal–oral route or *via* contaminated water or food (5). Additionally, *C. parvum* has an enormous capacity to reproduce in infected animals, by producing large amounts of infective oocysts being passed through the feces of infected animals. This leads to widespread contamination of grazing lands, water sources, and the general environment. Furthermore, it causes adverse consequences for productivity. Healthy and adult animals can also shed oocysts, often in large numbers, providing a potential reservoir of infection and environmental contamination (6). According to its clinical impact, cryptosporidiosis in cattle, among other species, is considered endemic worldwide and has been considered an important cause of scour in young, unweaned farm livestock, including calves, lambs, kids, alpacas, and foals. Mortality is generally low; however, mixed infection with other enteric pathogens, such as *Escherichia coli* or *Rotavirus*, has been reported. In contrast, severe outbreaks of cryptosporidiosis are sometimes reported (7). Some infected cattle exhibit reduced weight gain compared with uninfected controls, and one study has found that infection may interfere with milk production in dairy cows (6). Importantly, cryptosporidiosis should not only be considered from the perspective of animal health and production; its zoonotic characteristics and the possibility that animals act as a source of infection to humans *via* food stuff and water should also be considered. Although the infection leads to few deaths, serious economic losses can occur because of the costs involved in the treatment (8, 9).

In the available literature, the detection of *Cryptosporidium* oocysts in feces through microscopic examination is considered the most common way of determining infection (7). The parasites can be detected in fecal specimens; the results of this examination method can be enhanced by increasing the concentration of the samples (10). To increase the concentration of the samples, formol-ether (ethyl acetate) or flotation (often sucrose or sodium chloride) is usually used before microscopy. As the oocysts are minute, the use of a staining technique is recommended and considered the gold standard—the modified Ziehl–Neelsen (mZN) or auramine phenol staining technique has been used successfully (11). This method is low cost, can be used for screening numerous samples, and causes a permanent stain; therefore, doubtful or scanty positive slides may be sent to a reference laboratory for confirmation. Both hot and cold acid-fast staining methods are valuable as well (12). However, screening of positive samples using PCR-based techniques, such as nested PCR, which targets the amplification aspect of the sequence encoding 18S small subunit (SSU) rRNA, offers many advantages for fecal examination methods and might help confirm the infection (13, 14).

Providing periodical updates about the epidemiological data of the parasites through field surveys seems crucial for effectively implementing strategies for controlling the disease (15–19). In the available literature, little is known about the occurrence of cryptosporidiosis among cattle and buffalo calves in Upper Egypt and the real contributions of these animals in the maintenance of the epidemiological foci of the disease. Therefore, this study was designed to investigate the occurrence of cryptosporidiosis in buffalo and cattle calves in Upper Egypt through a parasitological study. Moreover, we confirmed positive samples from microscopy using molecular methods. The major individual variable factors that might be associated with the infection were also investigated.

## Materials and Methods

### Study Area, Sampling, Animal Data, and Clinical Examination

The present work involved a cross-sectional study that estimated the occurrence of cryptosporidiosis among cattle and buffalo calves admitted to public veterinary hospitals from different localities (*n* = 10) throughout Assiut Province, Egypt. The study was conducted during the period from April 2017 to June 2018. A random sampling design (20) was followed to select the target samples from the 10 localities in the studied area. At the time of the district visit, the main authors were responsible to collect fecal samples from animals of those small stakeholders in rural areas admitted to the veterinary services unit of each locality. The sample size was calculated as described elsewhere (20) using Win Episcope 2 based on a 95% confidence level, with an expected proportion of 10% of animals having cryptosporidiosis and an accepted error of 2.5%, with an unknown population size. The number of animals to be selected was estimated at 554 and this number was distrusted evenly between the 10 localities, and we decided to collect 60 animals from each locality, with the exception of one locality that was 68. Collectively, a total of 608 fecal samples were collected, including 324 samples from cattle (168 males and 156 females) and 284 samples from buffalos (124 males and 160 females). The samples were transported and examined in the Department of Parasitology, Faculty of Medicine, Assiut University, and Assiut Regional Lab (Animal Health Research Institute) to determine the presence of *Cryptosporidium* infection. For each animal, the sampling date, age, sex, and fecal consistency (diarrheic or formed) were recorded. Full details of the study cohort are shown in Table 1. This step also involved examination of the animals clinically to report any signs.

**Table 1:** The full details of the study cohort for each of the enrolled animal species, sex, age, condition of animals, and seasonal variation.

	**Species**		**Enrolled**
**Individual factor**	**examined**	**Criteria**	**animals**
Species	Cattle Buffalo	Cattle Buffalo	324
			284
Sex	Cattle Buffalo	M	168
		F	156
		M	124
		F	160
Age	Cattle Buffalo	<1 month	92
		1–3 months	188
		>3 months	44
		<1 month	84
		1–3 months	132
		>3 months	68
Condition of animals	Cattle Buffalo	Diarrheic	248
		Non-diarrheic	76
		Diarrheic	212
		Non-diarrheic	72
Seasonal variation	Cattle Buffalo	Spring	76
		Summer	92
		Autumn	80
		Winter	76
		Spring	80
		Summer	56
		Autumn	68
		Winter	80

### Preparation of the Fecal Samples

A fecal sample was obtained from each animal directly from the rectum using a sterile plastic glove; the sample was then placed in a plastic cup and transported to the laboratory into an icebox to be examined within 2–3 h. The collected samples were prepared and examined on the day of collection. The samples were transported and examined in the Department of Parasitology, Faculty of Medicine, Assiut University, and Assiut Regional Lab (Animal Health Research Institute) to determine the presence of *Cryptosporidium* infection.

### Macroscopic Examination of the Fecal Samples

The fecal samples were examined macroscopically to detect abnormalities in consistency and color, the presence or absence of blood, the state of digestion, and the presence of mucus or other unusual constituents according to the protocol described elsewhere (21).

### Parasitological Examination of the Fecal Samples

The fecal samples were filtered through two layers of gauze to remove the coarse particles and stored in an equal amount of 2.5% potassium dichromate solution at 4°C until the time of examination (22). All specimens were examined for *Cryptosporidium* oocysts under a microscope using a staining technique (23). The fecal samples were examined using the direct and saline smear methods according to the protocol described elsewhere (24).

### Direct Smear Method

This step was performed according to the protocol established elsewhere (25). Briefly, approximately 2mg of feces was mixed with a drop of physiological saline (0.85% NaCl) and placed on a clean slide. Then, the sample was thoroughly mixed until a uniform suspension was formed without fibers or gritty materials and covered with a 22 × 22mm cover glass until the sample was evenly spread. The examination was performed systematically and thoroughly using a 10× objective lens, and confirmation was made by switching to a magnification of 40×.

#### Simple Gravity Sedimentation Technique

Approximately 10 g of feces was thoroughly mixed with 50-ml of tap water in a 250-ml beaker. Then, the suspension was strained through two layers of wet gauze into a sedimentation flask, and the sample was allowed to sediment for 1 h. The supernatant was decanted carefully, the sediment was suspended by adding tap water, and the material was left to sediment for 1 h. Then, the supernatant was decanted carefully again. Washing was repeated until a clear supernatant was obtained. A small portion of the sediment was placed on a glass slide using a long capillary pipette. The slide was covered with a cover slide and examined for parasites (25).

#### Flotation Method

The fecal samples (~10 g) were placed in a cup and mixed thoroughly with ~50mL of tap water using a spatula. The mixture was poured through a wire mesh screen with an aperture of 500μm to remove large lumps. The strained fluid was caught in a bowl. The suspension was transferred to a conical measure and filled with tap water to the top and allowed to settle for 30 min. The supernatant was discarded carefully. The sediment was stirred, and a 2-ml sample was poured into a centrifuge tube. The tube was placed in a centrifuge. A saturated NaCl solution was added using a pipette until a convex meniscus stood above the top of the tube. A cover glass was placed on the tube, ensuring that no bubble was trapped under it. The tube was centrifuged at 2,000 rpm for 2min. The cover glass was removed, and the sample was placed on a slide and examined under a microscope (26).

#### Examination of the Fecal Samples Using the mZN Method

*Cryptosporidium* was directly identified using the modified Kinyoun acid-fast stain (cold method). The films were fixed in absolute methanol for 2min and allowed to dry. The slides were flooded with Kinyoun's Carbol-Fuchsin solution for 5min. The slides were rinsed briefly with 50% ethanol for 5 s, rinsed thoroughly with water, and decolorized with 1.5% sulfuric acid for 2min. Then, they were rinsed with water and drained, and counter-stained with methylene blue for 5min. The slides were rinsed again with water and allowed to air dry. The stained smears were systematically examined under a microscope at ×400 and ×1,000 magnification. *Cryptosporidium spp*. oocysts appear pink to red with spherical to ovoid bodies against a blue background (27).

### Genomic DNA Extraction

This step involved the extraction of DNA from *Cryptosporidium*-positive fecal samples identified using microscopy (*n* = 204 samples) using the QIAamp DNA MiniKit (Qiagen Co., USA) according to the manufacturer's protocol with slight modifications. Following the extraction, DNA concentrations were measured using DNA/Protein Analyzer (Quawell Q 9000, USA).

### Nested PCR Procedure

Nested PCR was performed as described by Nichols et al. (28). The two sets of PCR primers used in this study are listed in Table 2, and the expected amplicon size was 655–667 bp. The primary PCR reaction contained 2 μL of DNA in a 20-μL reaction volume, and the secondary PCR reamplified 5 μL of the primary PCR product. Non-acetylated bovine serum albumin (Invitrogen, USA) at a final concentration of 400 μg/mL was added to each PCR reaction. The primary and secondary protocols used were as follows: initial denaturation at 94°C for 5min; 40 cycles at 94°C for 1min, 55°C for 1min, and 72°C for 1min; extension at 72°C for 10min. PCR amplifications were processed using BIO-RAD Thermal Cycler (BIO-RAD, Singapore). After amplification, the PCR products were visualized in a 1% agarose gel stained with RedSafe Gel electrophoresis (Intron) electrophoresis using Molecular Imager (BIO-RAD, Singapore).

**Table 2:** Primers used in PCR.

**Primers**	**Sequence**
Primary forward	CPB-DIAGF: 5‘-AAGCTCGTAGTTGGATTTCTG-3‘
Primary reverse	CPB-DIAGR: 5‘-TAAGGTGCTGAAGGAGTAAGG-3‘
Secondary forward	N-DIAGF2: 5‘-CAATTGGAGGGCAAGTCTGGTGCCAGC-3‘
Secondary reverse	N-DIAGR2: 5‘-CCTTCCTATGTCTGGACCTGGTGAGT-3‘

### Statistical Analysis

Data were collected, tabulated, and statistically analyzed using SPSS computer programs version 16. CA hi-square test was used to compare qualitative variables. A probability level of *P* < 0.05 was chosen to indicate significant differences.

## Results

### Clinical Manifestations

The clinical signs observed on the animals under study were recorded. Some animals had diarrhea and other symptoms, such as debilitation, emaciation, dehydration, pale mucous membranes, and anemia.

### Occurrence of *Cryptosporidium* Infection in the Animals Under Study

**Figure 1:**
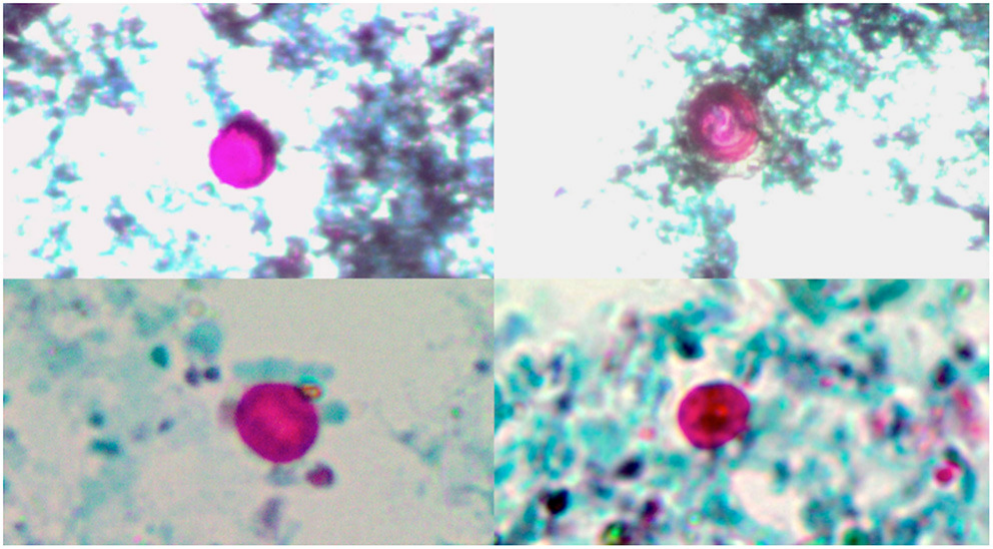
Fecal smears showing *Cryptosporidium* oocysts stained by modified Ziehl–Neelsen (1000X).

**Figure 2:**
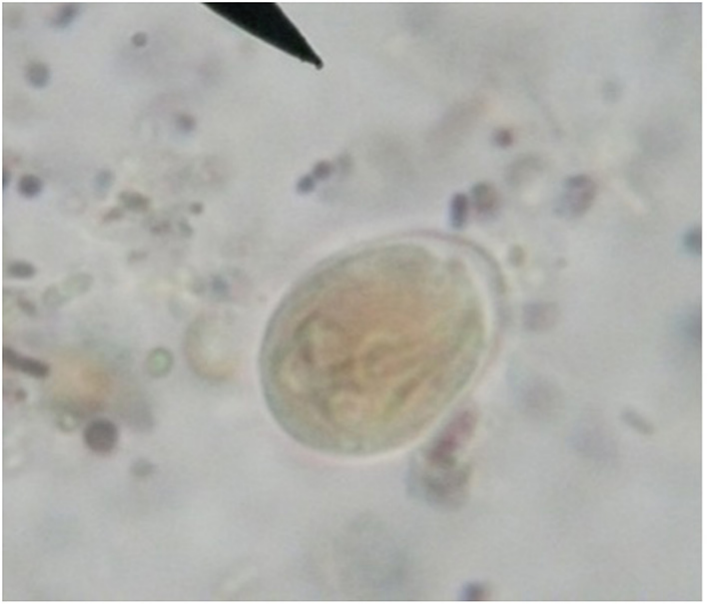
Fresh fecal smear showing *Giardia bovis* cyst (X40, Bar = 10μm).

**Figure 3:**
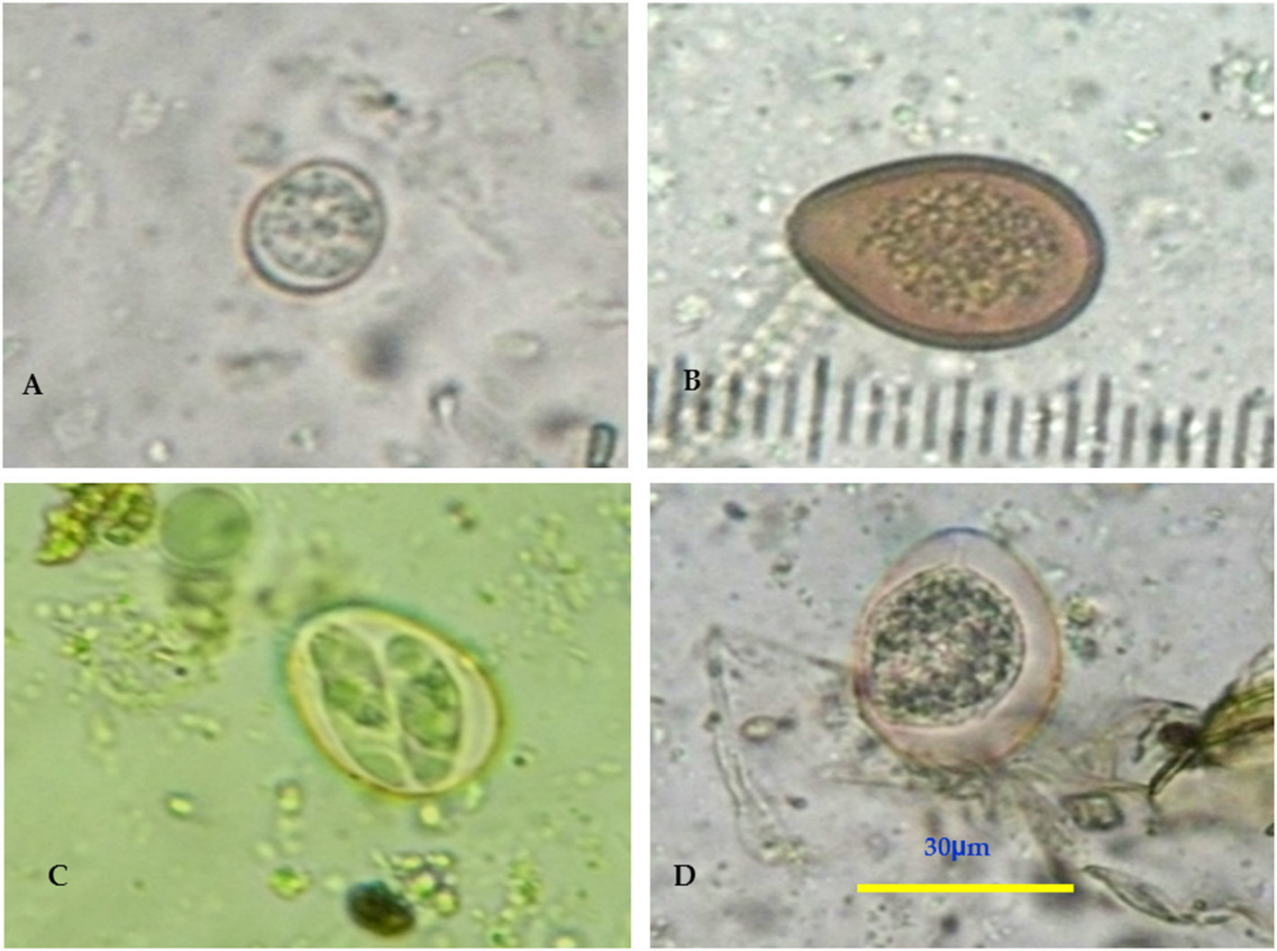
**(A–D)** Fresh fecal smear showing *Eimeria* spp. *oocyst* (X40, Bar = 30μm).

**Figure 4:**
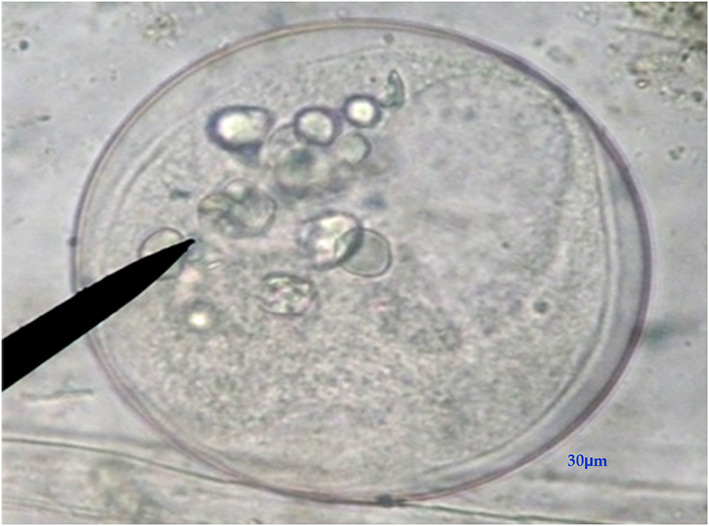
Fresh fecal smear showing *Buxtonella sulcata* cyst (X40, Bar = 30μm).

**Figure 5:**
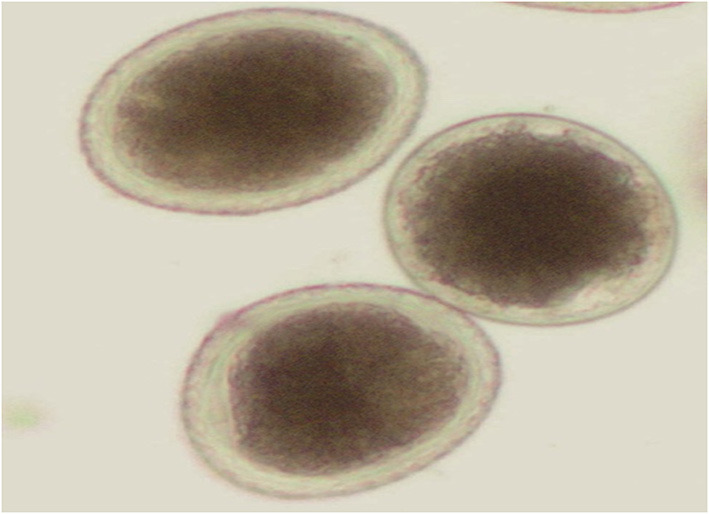
Fresh fecal smear showing *Toxocara vitiluram* egg (X40).

The parasitological examination revealed that ~33.55% (204/608) of the animals under study were infected with *Cryptosporidium*. Cattle calves had a higher infection rate (38.27%) than buffalo calves (28.16%) %) (Table 3). Furthermore, this study reported cases of mixed infection of *Cryptosporidium* spp. with different parasites in the cattle and buffalo calves under study during the parasitological examination. The major parasites detected are shown in Figures 1–5 and Tables 4, 5.

**Table 3:** Prevalence of *Cryptosporidium* infection in cattle and buffalo calves.

**Species**	**Number of examined**	**Number and percentage of infected**	**Positive rate%**	**Statistical data (*P*-value)**
Cattle	324	124	38.27	0.0627 (NS)
Buffalo	284	80	28.16	
Total	608	204	33.55	

*NS, No statistical significance differences (>0.05)*.

**Table 4:** Mixed infection of *Cryptosporidium* with different parasites in examined cattle & buffalo calves.

		***Cryptosporidium only***	***Cryptosporidium* protozoa +**	***Cryptosporidium* helminthes +**
Cattle (*n* = 324)	Number of infected	78	34	12
	Positive rate%	24.07	10.49	3.7
Buffalo (*n* = 284)	Number of infected	48	24	8
	Positive rate%	16.90	8.45	2.81

**Table 5:** Prevalence of different parasites in both cattle & buffalo calves.

**Parasite examined calves**		***Cryptosporidium***	***Eimeria***	***Giardia***	***Ascaris***	***Buxtonella***
Cattle (*n* = 324)	No. of infected	124	64	16	24	8
	%	38.27	19.75	3.08	9.23	3.64
Buffalo (*n* = 284)	No. of infected	80	92	12	16	–
	%	28.16	32.39	5.45	5.63	–
Total (*n* = 608)	No. of infected	204	156	20	40	8
	%	33.55	25.65	4.16	6.57	1.66

### Nested PCR

Interestingly, 202 DNA samples extracted from positive fecal samples provided the expected amplicon size (655–667 bp) in the nested PCR. However, two samples appeared negative.

### Epidemiological Data and the Potential Individual Variable Factors Associated With the Occurrence of Infection

Tables 6, 7 show the potential explanatory individual variable factors associated with the occurrence of infection with *Cryptosporidium* spp. in cattle and buffalo calves, respectively. According to cattle calves, the sex and condition of animals were the main variables that were significantly associated with the occurrence of *Cryptosporidium* spp. (*P* < 0.05). In this respect, in accordance with sex, there was a significant difference between males and females with a *p*-value (*p* < 0.001). As illustrated in Table 6, the highest positive rate was observed among female animals (51.28%). On the other hand, a positive rate in males was recorded (26.19%). Regarding the condition of animals, there was a statistically significant association between the frequency of positive samples and the condition of examined animals (diarrheic vs. non-diarrheic) as a potential risk factor (*p* < 0.0011). The majority of *Cryptosporidium* spp. were detected in diarrheic cattle calves (45.16%) than in non-diarrheic animals (15.78%). On the other hand, season and age were the variables that were not significantly associated with the occurrence of *Cryptosporidium* spp. In accordance with age as a potential individual variable factor, the occurrence of the infection in cattle calves aged >3 months 54.54% was higher than in the remaining age ranges. Regarding the potential influence of seasonal variation, the occurrence of infection was higher in spring and winter than in autumn and summer. In this context, a higher percentage of infected cattle calves was reported in spring (42.11%) and winter (52.63%) than in summer (30.43%) and autumn (30%).

**Table 6:** Summarizes the data of the possible associations between the occurrence of *Cryptosporidium* spp. in cattle calves and the potential explanatory individual variable factors of these detected species.

**Total number of examined animals**		**No. of positive**	**No. of negative**	**Positivity rate %**	***p*-value**
324		124	200	38.27	
**Variable factors**					
Age	No. of examined	Positive	Negative		0.2027
<1 month	92	36	56	39.13	
1–3 month	188	64	124	34.04	
>3 months	44	24	20	54.54	
Sex	No. of examined	Positive	Negative		0.0010^*^
Male	168	44	124	26.19	
Female	156	80	76	51.28	
Season	No. of examined	Positive	Negative		0.1162
Spring	76	32	44	42.11	
Summer	92	28	64	30.43	
Autumn	80	24	56	30	
Winter	76	40	36	52.63	
Condition of animals	No. of examined	Positive	Negative		0.0011^*^
Diarrheic	248	112	136	45.16	
Non-diarrheic	76	12	64	15.78	

* *Statistical significance differences (p <0.05)*.

**Table 7:** Summarizes the data of the possible associations between the occurrence of *Cryptosporidium* spp. in buffalo calves and the potential explanatory individual variable factors of these detected species.

**Total number of examined animals**		**No. of positive**	**No. of negative**	**Positivity rate %**	***p*-value**
284		80	204	28.16	
**Variable factors**					
Age	No. of Examined	Positive	Negative		0.9726
<1 month	84	24	60	28.57	
1–3 month	132	36	96	27.27	
>3 month	68	20	48	29.41	
Sex	No. of examined	Positive	Negative		0.3400
Male	124	40	84	32.25	
Female	160	40	120	25	
Season	No. of examined	Positive	Negative		0.5767
Spring	80	24	56	30	
Summer	56	12	44	21.42	
Autumn	68	16	52	23.52	
Winter	80	28	52	35	
Condition of animals	No. of examined	Positive	Negative		0.0085^*^
Diarrheic	212	72	140	33.96	
Non-diarrheic	72	8	64	11.11	

* *Statistical significance differences (p < 0.05)*.

According to buffalo calves, the condition of the examined animals was the only variable that was significantly associated with the occurrence of *Cryptosporidium* spp. (*P* < 0.0085). The remaining factors were not significantly associated with the occurrence of cryptosporidiosis. However, it could be noted that the association between the potential explanatory individual variable factors and the occurrence of infection in buffalo calves is in the same line of cattle calves with the exception of sex. In this regard, the highest positive rate was observed among male animals (32.25%), whereas the positive rate in females was recorded (25%). The higher percentages of infection were reported in those aged >3 months and in diarrheic buffalo calves 29.41 and 33.96%, respectively. In addition, the majority of *Cryptosporidium* spp. was detected in spring (30%) and winter (35%) than in autumn (23.52) and summer (21.42%).

## Discussion

This study provides interesting parasitological and molecular findings on the occurrence of *Cryptosporidium* infection and other related infections in cattle and buffalo calves in Assiut province, Upper Egypt. Moreover, this study explored the major epidemiological factors associated with the occurrence of infection. In the available literature, information about cryptosporidiosis in Upper Egypt is limited, and therefore, this study provides a novel contribution to the occurrence of this disease and the potential contribution of cattle and buffalos in the maintenance of the epidemiological foci of this zoonotic disease in this area. It should be stressed that the clinical signs of the infection can range from mild in older animals to severe in young animals; moreover, this infection can cause varying degrees of dehydration, dullness, anorexia, fever, and loss of condition. *Cryptosporidium-*infected buffalo calves showed yellowish, greenish, or clay-colored profuse watery diarrhea and reduced milk suckling, and with the progression of the disease, the animals were dehydrated with evidence of a lying down posture (7, 29). This study reports a series of clinical signs, including diarrhea, emaciation, dehydration, and pale mucous membranes. These findings agree with the results reported in a previous study (30). Note that the diagnosis of cryptosporidiosis usually depends on the demonstration of oocysts (or, less commonly, their antigens or DNA) in fecal samples. Moreover, antibody-based detection of oocysts in serum/plasma, saliva, or feces can demonstrate exposure to *Cryptosporidium*. However, it can only produce a diagnostic benefit if seroconversion can be demonstrated; otherwise, a positive result can indicate past exposure, current infection, or both (5). *Cryptosporidium* colonizes the mucosal surfaces of the affected organs; therefore, the easiest way to determine the infection status of *Cryptosporidium* is by microscopically examining epithelial cells of infected organs. Oocysts can be visualized using different histochemical stains, including Giemsa and Kinyoun's acid-fast stain (3, 31). Microscopic examination for detecting *Cryptosporidium* oocysts in feces is the most common way for confirming infection (7, 23). The detection of parasites in fecal specimens can be enhanced by increasing the concentration of the samples (7, 10). To increase the concentration of the samples, formol-ether (ethyl acetate) or flotation (often sucrose or sodium chloride) is usually used before microscopy. As the oocysts are minute, the use of a staining technique is recommended and considered the gold standard; the mZN or auramine phenol staining method has been used successfully (11). This study involved the surveillance of cryptosporidiosis infection among cattle and buffalo calves of different ages from different localities of Upper Egypt through microscopic examination and modified Kinyoun's acid-fast staining, which is consistent with several previous studies (32).

In this study, the overall occurrence of infection with *Cryptosporidium* spp. among the cattle and buffalo calves under study was 33.55%; moreover, the percentage of infection was more pervasive in cattle (38.27%) than in buffalos (28.16%), though no statistically significant difference (*p* < .05) was observed. In the available literature, lower occurrence rates of infection were reported at either the national or international level. A study (33) has reported a prevalence rate of 14.2% *Cryptosporidium*in Dakahlia and Kafr El-Sheik Governorates (Middle Egypt). Furthermore, (34) have reported an overall prevalence of 13.6% in dairy cattle in the Nile River delta provinces, Egypt. Another study (20) investigated the occurrence of infection in cattle and buffalos in Beni-Suef Governorate (Egypt) and reported a prevalence rate of 10.2 and 12.3%, respectively. Likewise, (35) revealed that 19.2 and 20.4% of the cattle and buffalos under study, respectively, were infected in Ismailia province (Egypt). Higher results were reported in a previous study (36) in Behera province (Egypt) where the parasite was detected in 35 and 52.9% of the examined cattle and buffalos, respectively. In contrast, nearly similar results were reported in a previous study (37) in Mumbai, India, where the parasite was detected in 36.99 and 34.48% of the examined buffalos and cattle, respectively. Another study (38) has revealed a prevalence rate of 17.65 and 6.25% in buffalos and cattle, respectively, from India. The variations in the prevalence rates of the disease between cattle and buffalo calves may be attributed to various factors, including the geographical location, sample size, seasonal variation, environmental climatic conditions, a system of rearing and management, and the level of hygienic measures applied (39). According to the molecular identification, it should be stressed that the 18S SSU rRNA gene *locus* is widely used as a molecular marker for molecular detection and characterization of *Cryptosporidium* isolates from humans and various animals (40). As shown in the results of this study, nested PCR yielded amplicons with the expected product size of 655–667 bp in almost all fecal samples using microscopy (202/204). Furthermore, two samples provided negative results using PCR; however, they were positive for *Cryptosporidium* using microscopy. The possible explanation is the failure of oocyst disruption and DNA extraction or that the concentration of oocysts in the samples subjected to extraction was insufficient. This finding coincides with those reported in a previous study (41), which reported that five samples were positive in the microscopic examination of stained fecal smears; however, they were negative on PCR. Our results are also consistent with another previous study reported that the use of a poor DNA extraction method or demonstrating parasite DNA degradation in stool samples or the presence of inhibitors of PCR that might result in lower sensitivity of the PCR method than microscopy and is associated with overdiagnosis (42).

According to the potential explanatory variable factors, the statistical analysis revealed that age and seasonal variation were not significantly associated with the occurrence of *Cryptosporidium* spp. infection in buffalos or cattle. In this study, although we recorded different prevalence rates for different age groups, these differences were not statistically significant (*p* > 0.05). However, a previous study (43) revealed a significant association between the occurrence of infection and the age of the animals examined, with the highest prevalence recorded in young calves. In another study, infection was reported in all age groups; however, the infection was most common in pre-weaned calves. In contrast, when the calves reached the age of 2 months, 5–93% of them shed oocysts (44). The data presented in this study revealed that the infection rates in cattle calves more than 3, 1–3, and <1 month old were 54.54, 34.04, and 39.13%, respectively, whereas the corresponding percentages in buffalo calves were 29.41, 27.27, and 28.57%, respectively. These results are relatively variable than those previously reported in Behera province (Egypt), where the prevalence rates were 41.4 and 44.4% in 1–4-week-old and 4–8-week-old calves, respectively (36). A study (37) conducted a survey in Mumbai, India, and reported a positivity rate of 38.4 and 10% among cattle calves <3 months old and adult animals, respectively, and 40.23 and 6.25% in buffalo calves and adult ones, respectively. Another study (35) in Ismailia province (Egypt) reported that the prevalence rates were as follows: 30.3% in cattle calves aged 1 day to 3 months, 13.3% in those aged >3 months to 1 year, 12.5% in those aged >1 to 2 years, and 5.2% in those aged >2 years and 42.9% in buffalo calves aged 1 day to 3 months, 30.1% in those aged >3 months to 1 year, 25.9% in those aged >1 to 2 years, and 15.9% in those aged >2 years. Furthermore, (45) microscopically identified *Cryptosporidium* oocysts in 4.17% of fecal samples obtained from water buffalo calves and in 0.48% of adult animals, whereas no oocysts were detected in specimens obtained from heifers. The differences in the reported infection rates may be attributed to sampling size, the season of specimen collection, the system of rearing and management, the level of hygienic measures applied, environmental factors, and management and husbandry regimens used (45).

As shown in our results, the occurrence of cryptosporidiosis in female cattle was significantly higher than in male animals (*p* < 0.001). However, no statistically significant difference in the frequency of *Cryptosporidium* infection in buffalos in terms of sex (*p* > 0.05). In this study, infection with *Cryptosporidium* spp. was more predominant in female cattle calves (51.28%) than in males (26.19%); in contrast, male buffalo calves (32.25%) were more susceptible than females (25%). A study (20) in Beni-Suef Governorate (Egypt) reported that the infection rates in male and female cattle calves were 12.4 and 9.1%, respectively, whereas the prevalence rates in male and female buffalo calves were 14.5 and 10.7%, respectively. Additionally, another study (36) examined the occurrence of infection in cattle and buffalo calves in Behera province (Egypt) and found a positivity rate of 45.5 and 36.8% in male and female animals, respectively. Another study in Egypt (46) found that the infection rate was 12.69% in male dairy cattle calves and 12.3% in female ones. Furthermore, (47) in Addis Ababa (Ethiopia) examined the occurrence of infection in cattle calves and found that the occurrence of the disease was 13.7% and 21.3% in males and females, respectively. The higher infection rates in female cattle than in male animals might be related to the management practices because males are moved too far for grazing and watering, whereas females are usually managed around homesteads (48). In contrast, males are fattened for a short period indoors and are fed mainly on dry rations until their slaughter, which reduces the chance of contracting the infection (49). As most dairy complexes used to cull male calves soon after birth, the number of fecal samples collected from male calves was lesser than from female calves, and most farmers rear more females than males. The lower infection rate in females than in males might be also attributed to the protective immunity, which develops early in life for most females. This immunity regulates the parasite population, whereas males develop the immunity more slowly (50).

Concerning the association between seasonal variations and the occurrence of *Cryptosporidium* spp. infection in cattle calves, this study revealed an infection rate of 52.63% in winter and 42.11% in spring, whereas the occurrence was 30.43 and 30% in summer and autumn, respectively. The infection rate in buffalo calves was 35% in winter, 30% in spring, and 23.52% in autumn, and the lowest infection rate was in summer (21.42%). However, as shown in this study, no significant differences in the infection rate according to the season were observed between cattle and buffalo calves (*p* >.05). These results agree with the findings reported in a previous study (30) in Egypt, which recorded a high infection rate in cattle calves in winter (39.9%) and a low infection rate in summer (24.7%). However, this study disagrees with a previous study (51), which reported a high prevalence of infection in cattle in summer (15%) and a low prevalence in winter (6.6%); meanwhile, the prevalence rates in buffalo calves were 20% during summer, 12% in autumn, and 7.5% in spring, and the lowest rate was in winter (4.5%). Bhat et al. (52) reported that infection in cattle calves in the Ludhiana District of Punjab, India, peaked during the monsoon season (40.35%), followed by summer (39.65%). In the same study, the lowest occurrence of 34.04% was recorded during the winter season (52). Clearly, these findings suggest that the prevalence of infection with *Cryptosporidium spp*. is not only related to the presence of calves at risk but also related to the presence of the suitable climatic conditions, which influence the viability and longevity of oocysts, and therefore, this has a direct bearing on the level of environmental contamination and source of infection. It seems that the temperature of the region under study in winter is suitable for the viability and survival of *Cryptosporidium* oocysts, which might result from the greatest contact with the source of infection, favoring the transmission of infection.

In the present work, significant differences in animal conditions were observed between cattle and buffalo calves [(*p* < 0.0011 and *p* < 0.0085), respectively]. In this respect, the occurrence of *Cryptosporidium* infection was higher in diarrheic cattle calves (45.16%) than in non-diarrheic ones (15.78%) and in diarrheic buffalo calves (33.96%) than in non-diarrheic ones (11.11%). This result agrees with those reported in several studies, which reported a higher prevalence of cryptosporidiosis in diarrheic cattle calves than in non-diarrheic animals (20, 32, 53, 54). The observations of this study suggest that at young ages, diarrhea could be caused by *Cryptosporidium* in both cattle and buffalos. An important finding of this study was the mixed infections with other enteric parasites, that is, *Eimeria spp. Toxocara vitulorum, Buxtonella sulcate*, and *Giardia* spp. In this respect, *Cryptosporidium* was associated with protozoa (i.e., *Eimeria spp., Buxtonella sulcate, and Giardia spp*.) in 10.49% of the calves examined, and mixed infections with helminths (*Toxocara vitulorum*) was observed in 3.7% of the cattle calves examined. Meanwhile, this study revealed that the occurrence of *Cryptosporidium* infection was associated with protozoa in buffalo calves (8.45%) and helminths in cattle calves (2.81%). In contrast, the occurrence of *Cryptosporidium* infection alone was reported in 24.07 and 16.90% of the cattle and buffalo calves examined, respectively. A previous study reported that *C. parvum*, together with *Giardia intestinalis*, was detected in 2% of the calves examined from dairy herds in southeast Sweden (55). Additionally, a study in India (56) reported that C. *parvum* alone was found in only 10% of the fecal samples obtained from diarrheic calves, whereas 90% of the samples showed mixed infections with the following agents: *Rotavirus, Coronavirus, Salmonella, E. coli*, and *Eimeria* spp. It seems that these different enteropathogens might enhance the susceptibility of newborn animals to *Cryptosporidium* infection.

## Conclusions

Given the aforementioned information, this study demonstrated a high occurrence rate of *Cryptosporidium* spp. infection among buffalo and cattle calves in Upper Egypt. Moreover, our parasitological, epidemiological, and molecular data concluded that buffalo and cattle play a potential role in the maintenance of the transmission cycle of the parasite in upper Egypt. Further molecular research and epidemiological studies are recommended to explore the involvement of other intermediate hosts in Egypt at a large-scale level and genotypic identification of the species of the parasites that are circulating throughout the country, which is important for combating infections caused by these organisms.

## Data Availability Statement

The original contributions presented in the study are included in the article/supplementary material, further inquiries can be directed to the corresponding author/s.

## Author Contributions

EKE, HS, SA-R, HB, MIA, and AG were involved in the conception of the research idea and methodology design, supervision, and performed data analysis and interpretation. MAA, DA, AA, EE, EAAH, and FE-G participated in methodology, sampling, data analysis, and contributed their scientific advice. EKE, HS, and AG drafted and prepared the manuscript for publication and revision. All authors read and approved the final manuscript.

## Conflict of Interest

The authors declare that the research was conducted in the absence of any commercial or financial relationships that could be construed as a potential conflict of interest.

## Publisher's Note

All claims expressed in this article are solely those of the authors and do not necessarily represent those of their affiliated organizations, or those of the publisher, the editors and the reviewers. Any product that may be evaluated in this article, or claim that may be made by its manufacturer, is not guaranteed or endorsed by the publisher.
